# Effect of Ultraviolet Light Irradiation Combined with Riboflavin on Different Bacterial Pathogens from Ocular Surface Infection

**DOI:** 10.1155/2017/3057329

**Published:** 2017-10-12

**Authors:** Jing Shen, Qingfeng Liang, Guanyu Su, Yang Zhang, Zhiqun Wang, Hong Liang, Christophe Baudouin, Antoine Labbé

**Affiliations:** ^1^Beijing Institute of Ophthalmology, Beijing Tongren Eye Center, Beijing Tongren Hospital, Capital Medical University and Beijing Ophthalmology & Visual Sciences Key Laboratory, National Engineering Research Center for Ophthalmology, Beijing 100005, China; ^2^INSERM, U968 and UMR_S 968, Institut de la Vision, University Paris 06 (UPMC) and CNRS, UMR_7210, 75012 Paris, France; ^3^Quinze-Vingts National Ophthalmology Hospital, Paris and Versailles Saint-Quentin-en-Yvelines University, Versailles, France

## Abstract

In order to study* Staphylococcus epidermis* and* Staphylococcus aureus in vitro* viability after the exposure to ultraviolet (UV) light and riboflavin, twelve strains of* Staphylococcus epidermis *and twelve strains of* Staphylococcus aureus *were isolated from patients with bacterial keratitis. The growth situation of* Staphylococcus epidermidis* and* Staphylococcus aureus* under different experimental conditions was qualitatively observed. The number of colonies surviving bacteria was counted under different UV light power and different exposure time. The experiment showed that there was no inhibition effect on the growth of bacteria using riboflavin alone. In UV alone group and UV-riboflavin group, inhibition effect on the bacteria growth was found. The UV-riboflavin combination had better inhibition effect on bacteria than UV irradiation alone. The amount of bacteria in the UV-riboflavin group was decreased by 99.1%~99.5% and 54.8%~64.6% in the UV alone group, when the UV light power was 10.052 mW/cm^2^ and the irradiation time was 30 min. Moreover, with the increase of the UV power or irradiation time, the survival rates of bacteria were rapidly reduced. Compared with* Staphylococcus aureus*,* Staphylococcus epidermis *was more easily to be killed under the action of UV light combined with riboflavin.

## 1. Introduction

Infectious keratitis is a vision-threatening condition, which can be caused by bacteria, virus, fungus, parasites, and so forth. It was estimated that 6 million infectious corneal ulcers occurred annually in the 10 countries of southeast Asia with a total population of 1.6 billion [1]. In China, there are 3 million corneal blind patients, and infectious corneal blindness has an annual increase rate of 100,000 cases [2]. Bacterial keratitis is one of the most common ocular infections, which progresses rapidly and severely. If the appropriate antimicrobial treatment is delayed, it can lead to devastating complications, including blindness and loss of the eye. However, with the extensive use of broad spectrum antibiotic, recent studies have shown increasing evidence of resistance of microbes to antimicrobial agents. Antibiotic resistance can cause continued progression of the disease process despite the use of broad spectrum antibiotics and some more severe consequences [3–5]. The occurrence of antibiotic-resistant bacteria has prompted ophthalmologists to study the antimicrobial activity of additional biological, chemical, and physical sources as adjunctive or alternative therapies for bacterial keratitis.

Riboflavin and UV light-induced corneal collagen cross-linking (CXL) is a therapeutic procedure used in the visual sciences which is based on irradiation of the corneal surface with UV-A light (370 nm) in combination with the administration of riboflavin (vitamin B2) to increase the biomechanical strength of collagen fibrils of the cornea. The main objective of the CXL technique was initially to avoid the progression of keratoconus [6, 7]. In some cases, CXL has also been proposed to treat infectious keratitis [8–10]. CXL is also one kind of photodynamic therapy (PDT) methods. PDT is a technique that utilizes reactive oxygen species (ROS) produced by a nontoxic dye or photosensitizer (PS) molecule in the presence of low intensity UV light or visible light to kill microbial cells. The PS molecule was excited from the ground state through the excited singlet state to a triplet state. Then, in the presence of oxygen, the PS undergoes reactions that produce reactive oxygen species to induce cell damage via oxidative stress [11–13]. Since the PS localizes to certain cells, only target cells in the irradiated area are damaged. Drug-resistant bacteria can be effectively eliminated by PDT [14] and there are no reports of microbes becoming resistant to PDT despite numerous attempts to induce resistance by repeated cycles of semilethal PDT and microbial regrowth [15].

In this study, we evaluated* in vitro* bactericidal effect of PDT on common ocular surface pathogens,* Staphylococcus epidermis* and* Staphylococcus aureus*. UV-riboflavin combination was selected to be used. The aim of this study is to develop a possible adjunctive or alternative therapy for bacterial keratitis.

## 2. Materials and Methods

### 2.1. Materials

#### 2.1.1. Bacteria Isolation and Culture

Twelve isolates of* Staphylococcus epidermis* and 12 isolates of* Staphylococcus aureus* were selected for this study. All bacterial strains isolated from patients with bacterial keratitis were provided by the Department of Microbiology, Beijing Institute of Ophthalmology, Beijing Tongren Hospital. The bacterial strains stored in the glycerol tube were inoculated in a blood culture dish and resuscitated two times to a logarithmic growth phase. Then the bacterial colonies were scraped with an inoculation loop and placed into the bacterial diluents. After sufficient mixing, 0.5 McFarland (MCF) turbidity standard bacterial suspensions were prepared using a turbid-meter (approximate bacteria concentration of 1~2 × 10^8^ colony-forming units/ml, CFU/ml), which was used for the subsequent experiments.

#### 2.1.2. Ultraviolet Light Source and Photosensitizer

The ultraviolet (UV) irradiation was performed with the LED point source (LAMPLIC UVEC-4, Blue Spectrum Rick Technology Co., Ltd., Shenzhen, China) with a wavelength of 370 nm. Calibration of the light energy was carried out before each experiment to ensure that the output power density was in range of 0.58 mW/cm^2^~10.052 mW/cm^2^. The light power density was measured by optical power meter (VLP-2000, Femtosecond Technology Co., Ltd., Changchun, China). The diameter of light spot was about 7 mm. In this experiment, the PS was riboflavin (Sigma-Aldrich Technology Co., Ltd., USA) which was dissolved into 2.5% wt solution of riboflavin with sterile phosphate buffered saline (PBS) and stored at 4°C in dark area.

### 2.2. Methods

#### 2.2.1. Qualitative Observation of the Inhibition Effect on Bacteria* In Vitro*

**Figure 1:**
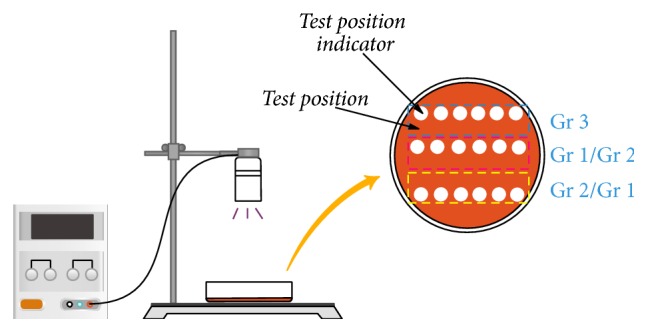
The experiment setup (Gr 1: UV-riboflavin group; Gr 2: control treated with UV alone group; Gr 3: control treated with riboflavin alone group).

Stock solution of riboflavin, 2.5% wt, was diluted to 0.1% wt with sterile PBS. In this experiment, four different samples were prepared which are UV-riboflavin treated sample (Group 1), control treated with UV alone (Group 2), control treated with riboflavin alone (Group 3), and control untreated. 200 *μ*L bacterial suspension (0.5 MCF) was aseptically transferred into the 9 cm blood agar plates with properly labeled test positions of control samples and UV-riboflavin treated sample. 10 *μ*L diluent riboflavin solution (0.1% wt) was added in each labeled test position of UV-riboflavin treated sample and riboflavin alone treated control sample. Before exposure to UV light, the samples to be irradiated were incubated in dark for 20 min. 370 nm UV light was used to irradiate UV-riboflavin treated sample and UV alone treated sample. The samples were irradiated by different power of UV light (10.052 mW/cm^2^, 7.299 mW/cm^2^, 5.273 mW/cm^2^, 2.474 mW/cm^2^, 1.065 mW/cm^2^, and 0.58 mW/cm^2^, resp.) for 20 min. Also irradiation time of 2 min, 5 min, 7 min, 10 min, 15 min, 20 min, and 30 min was taken for* Staphylococcus epidermis *and* Staphylococcus aureus* when the light power density was fixed at 5.273 mW/cm^2^. The entire experimentation was carried out in the dark box to avoid the influence of the ambient light. After irradiation, the experimental samples were incubated at 37°C for 48 hours. And then the growth situation of bacteria in different groups was observed and recorded. The detailed experiment setup is shown in Figure 1.

#### 2.2.2. Bacteria Inactivation Experiments* In Vitro*

*(1) Inhibition Effect of Different UV Light Energy*. 0.5 MCF bacteria suspensions of* Staphylococcus epidermis* and* Staphylococcus aureus* diluted into 1 : 10 with PBS were taken as UV alone group samples. 100 *μ*L bacteria suspensions (0.5 MCF) and 400 *μ*L riboflavin solution (2.5% wt) were taken into 500 *μ*L PBS as UV-riboflavin group samples. The initial population densities of experimental samples were both maintained at about 10^7^ CFU/ml and the concentration of riboflavin solution was about 0.1% wt.

150 *μ*L aliquots of the sample solutions were subjected to 96-well culture plates (Costar Corning, New York, USA). The inside diameter of the well is about 5 mm. The distance of light source to surface of solution was 5 mm. And the light spot diameter was about 7 mm at this distance, which was larger than the well and enabled satisfying irradiation of suspension surface with UV light. The illumination was conducted in dark box to prevent photosensitization of riboflavin from background visible light. The energy density was 1.065 mW/cm^2^, 2.474 mW/cm^2^, 5.273 mW/cm^2^, and 7.299 mW/cm^2^, respectively, and the irradiation time was 20 min. For the control group samples, the suspensions in 96-well plates were kept in the dark at room temperature for 20 min.

*(2) Inhibition Effect of Different Irradiation Time*. The experimental samples were prepared as described above (see Section 2.2.2(1)). In this part of experiment, the UV light energy was chosen as 5.273 mW/cm^2^. The irradiation time was 5 min, 10 min, 20 min, and 30 min, respectively. After the experiment, the counting of the number of bacterial colonies and the calculation of survival fractions were conducted as mentioned below.

*(3) Cell Concentration Determination and Survival Fraction Calculation*. After the experimental treatments, aliquots (100 *μ*L) were withdrawn from each well and serially diluted 10-fold with PBS. Ten microlitres from each dilution mixture was streaked onto blood agar plates (Tianjin Jinzhang Technique Development Co., China) in triplicate [16]. After incubation for 48 hours at 37°C, bacterial colonies were counted.

The survival fraction was calculated according to the equation *N*/No., where No. is the number of CFU per mL of bacteria without being treated and *N* is the number of CFU per mL of bacteria treated with UV light and riboflavin. All results were presented as means ± standard deviation (SD) of at least three independent experiments and each was measured in triplicate.

### 2.3. Statistical Analysis

Statistical analyses were performed by SPSS software (version 18.0, SPSS Inc., Chicago, Illinois, USA). Experimental data were confirmed to be of normal distribution by the* K*-*S* test. Descriptive statistics were used to summarize the data in multiplex analyses. The results were shown as the means ± SD. The statistical significance between groups was determined using two-way analysis of variance (ANOVA). *P* < 0.05 were considered to be statistically significant.

## 3. Results

### 3.1. Qualitative Observation of the Inhibition Effect on Bacteria

**Figure 2:**
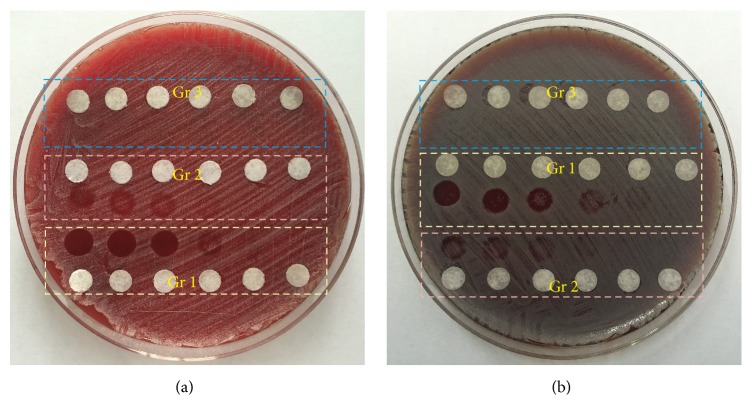
The bactericidal effect under the same irradiation time (20 min) and different light power ((a)* Staphylococcus epidermis*, (b)* Staphylococcus aureus*). The power density of UV light from left to right was 10.052 mW/cm^2^, 7.299 mW/cm^2^, 5.273 mW/cm^2^, 2.447 mW/cm^2^, 1.065 mW/cm^2^, and 0.58 mW/cm^2^. White paper used to mark the location of the test site. (Gr 1: UV-riboflavin group; Gr 2: control treated with UV alone group; Gr 3: control treated with riboflavin alone group.)

**Figure 3:**
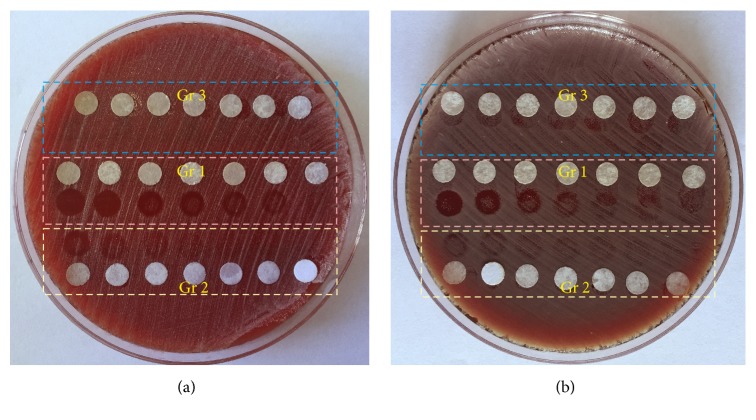
The bactericidal effect under the same light power density (5.273 mW/cm^2^) and different irradiation time ((a)* Staphylococcus epidermis*, (b)* Staphylococcus aureus*). The irradiation time of UV light from left to right was 30 min, 20 min, 15 min, 10 min, 7 min, 5 min, and 2 min. White paper used to mark the location of the test site. (Gr 1: UV-riboflavin group; Gr 2: control treated with UV alone group; Gr 3: control treated with riboflavin alone group.)

The growth situation of* Staphylococcus epidermidis* and* Staphylococcus aureus* under different experimental conditions is shown in Figures 2 and 3.  Figure 2(a) shows that the growth situation of* Staphylococcus epidermidis *in different experimental groups changed with different light energy irradiated.  Figure 2(b) shows the changes in bacterial growth of* Staphylococcus aureus *in different experimental groups exposed to UV light with different energy. The irradiation time is settled as 20 min for both of the bacterial species. Obviously, there is no inhibitory effect on the growth of bacteria in the control treated with riboflavin alone groups for both kinds of bacteria. The number of bacteria in the control treated with UV group decreases gradually with the increase of the UV light energy. However, UV-riboflavin treated group shows significant decrease in the viability when compared to all other control samples. Effect of bacterial killing is more pronounced with the increase of UV light energy in both of these bacterial species. Compared with Group 2, fewer bacteria survive in Group 1 exposed on UV light with the same energy. As shown in Figure 2(a), when the light power is greater than or equal to 5.273 mW/cm^2^, the bacteria could not continue to grow with the presence of riboflavin, indicating that all the bacteria are inactivated.

Figure 2(b) shows the growth of* Staphylococcus aureus* under different experimental conditions, which is similar to that of* Staphylococcus epidermidis*. In Group 3, no bacteriostatic region is produced on the medium, and the bacteria grow normally as control untreated group. There is an obvious reduction of the bacteria number in Group 1 and Group 2. Moreover, the survival of bacteria in the light area became less with the increase of UV intensity. By comparison of Group 1 and Group 2, it is also found that more bacteria were killed at the same light power irradiation when the riboflavin is present. However, to achieve the same efficacy of killing,* Staphylococcus aureus* required higher light energy than* Staphylococcus epidermidis*, as shown in Figures 2(a) and 2(b).

Figure 3(a) shows that the growth situation of* Staphylococcus epidermidis* in different experimental groups changed with the irradiation time. The changes in bacterial growth of* Staphylococcus aureus* in different experimental groups after being exposed to UV light for different time were shown in Figure 3(b). In this part of experiment, the power density of UV light is kept with 5.273 mW/cm^2^. As shown in Figures 3(a) and 3(b), the bacteria are in normal growth for both of the bacterial species in Group 3. UV-riboflavin treated groups in both bacterial species show significant decrease in the viability when compared with the control group treated with UV only at the same irradiation time. Moreover, along with the extended irradiation time, the bacteria-killing effect is improved significantly. However, compared to* Staphylococcus epidermidis, Staphylococcus aureus* is more difficult to eliminate under the same experimental condition. In order to kill the same amount of bacteria,* Staphylococcus aureus* needs more time to be exposed with the UV light.

### 3.2. Quantitive Analysis of the Inhibition Effect on Bacteria

**Figure 4:**
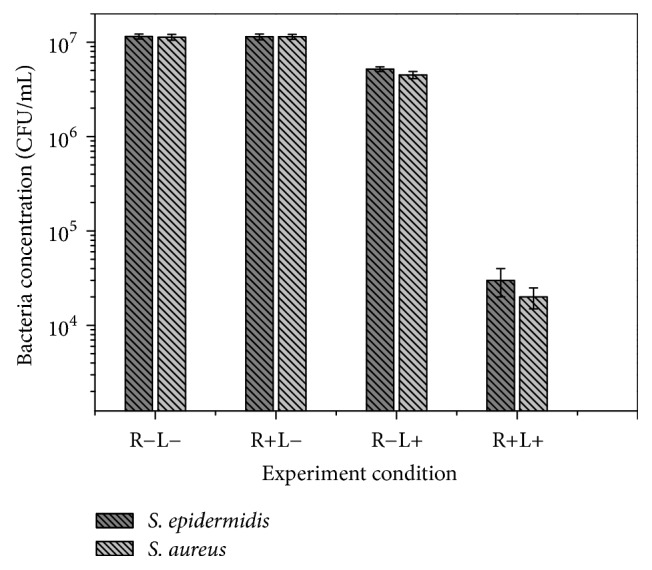
The number of survival bacterial colonies after different bactericidal experiment (R: riboflavin; L: UV light) to* Staphylococcus aureus* and* Staphylococcus epidermis* (light power density: 10.052 mW/cm^2^, irradiation time: 30 min).

The quantitive comparison test was conducted in the four experimental groups. The power density of UV light was 10.052 mW/cm^2^ and the irradiation time was 30 min. The concentration of riboflavin solution was about 0.1% wt. The results in Figure 4 showed that the colony number of* Staphylococcus epidermidis and Staphylococcus aureus* in different experimental groups was statistically significant. The colony number of the two bacteria in the control treated with UV alone group was significantly lower than that in the control untreated group and the difference was statistically significant (*P* < 0.05). The colony number of the two bacteria in the UV-riboflavin treated group was also significantly lower than that in the control untreated group (*P* < 0.05). Compared with the control untreated group, the amount of bacteria in the control treated with UV alone group decreased by 54.8%~64.6%, and the amount of bacteria in the UV-riboflavin treated group decreased by 99.1%~99.5%. There was no significant difference in the amount of bacteria between the control treated with riboflavin only and the normal control untreated group in all bacterial strains (*P* > 0.05).

**Figure 5:**
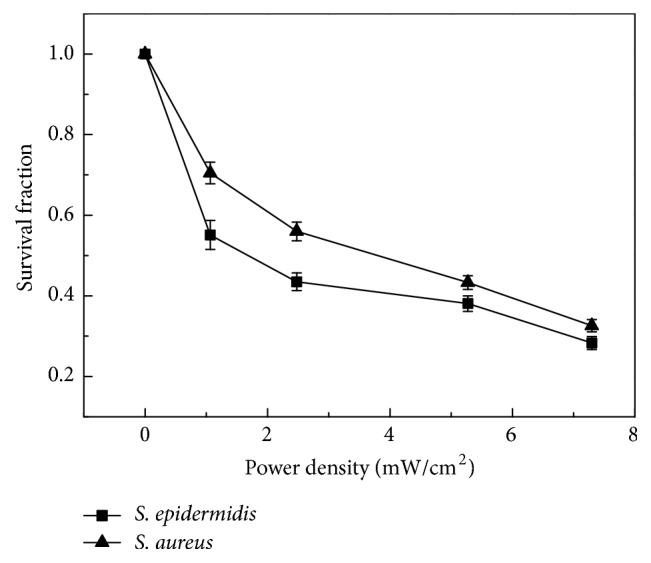
Survival fraction of* Staphylococcus epidermis* and* Staphylococcus aureus *with different UV power in the UV-riboflavin group (irradiation time: 20 min).

In this portion of the study, PDT was conducted under the varying conditions in order to further understand the bactericidal efficacy. The survival fractions of* Staphylococcus epidermis* and* Staphylococcus aureus *under different UV light power were calculated and shown in Figure 5. With the increase of the UV light power, the survival fractions of both of the bacteria were rapidly reduced. However, it is clear that the survival fraction of* Staphylococcus epidermis* is lower than that of* Staphylococcus aureus *using the same UV light power. When the UV light power was 1.065 mW/cm^2^, about 50% of* Staphylococcus epidermis* was killed after 20 min irradiation, but 70% of* Staphylococcus aureus* still survived. With further increase in light power, the survival fraction of the two bacteria decreased correspondingly, but the difference between them still existed. With the increase of UV light power, the difference of the survival fraction between* them *became smaller. The details of survival fraction of* Staphylococcus epidermis *and* Staphylococcus aureus *under different UV light power were listed in Table 1.

**Figure 6:**
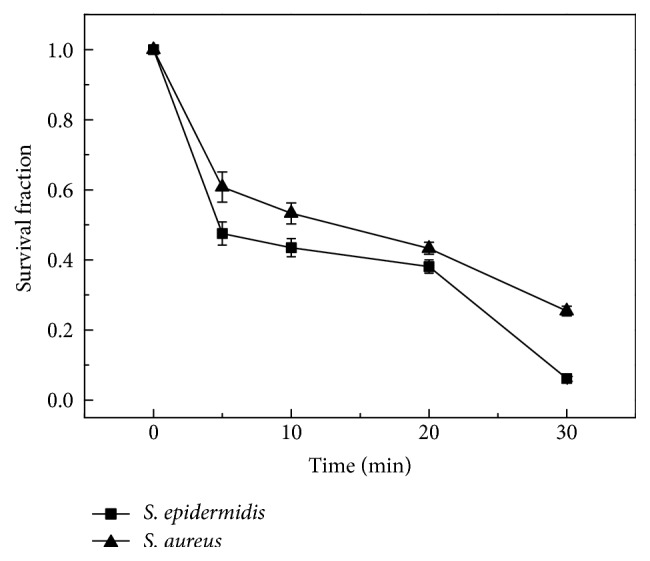
Survival fraction of* Staphylococcus epidermis* and* Staphylococcus aureus* under different UV irradiation time in the UV-riboflavin group (light power density: 5.273 mW/cm^2^).

The survival fractions of* Staphylococcus epidermis* and* Staphylococcus aureus *with different irradiation time were calculated and shown in Figure 6. The survival fractions for both of the bacteria were rapidly reduced with extending the irritation time. Even with only 5 min irradiation, the survival fraction of* Staphylococcus epidermis* reduced to 45%, and the survival fraction of* Staphylococcus aureus *reduced to 60%. There was still difference in the survival fraction between the two kinds of bacteria. Compared with* Staphylococcus aureus*,* Staphylococcus epidermis *was more easily to be killed under the action of UV light combined with riboflavin. The details of survival fraction of* Staphylococcus epidermis *and* Staphylococcus aureus *with different time irradiation were also listed in Table 2.

## 4. Discussions

The UV light as a therapeutic tool has been found around for thousands of years. Raab used PDT for antimicrobial studies more than 100 years ago, but this kind of research was not to be continued and the focus had shifted to its antitumor mechanisms and clinical applications [17–19]. Oppositely, the progress of PDT for antimicrobial treatment was very slow. In recent years, along with the emergence of drug-resistant bacteria, the researchers began to reexamine the value of PDT in local resistance to infection [20].

In the last decade, the research of PDT antimicrobial effects was launched and has made more progress. At the beginning, the studies focused on the disinfection of whole blood and blood components [21]. The present study found that PDT can effectively kill multidrug-resistant strains, which is even more effective than some antibiotics. Moreover, bacteria were difficult to produce resistance to PDT. From our study, the amount of bacteria in the UV-riboflavin group was decreased by 99.1%~99.5%, and the bacterial count was decreased by 54.8%~64.6% in the control UV alone group. With the increase of the UV intensity, the survival rates of bacteria were rapidly reduced. The photosensitizer, like riboflavin, could be used to inactivate bacteria combined with UV light, whose mechanism was involved in the cytotoxic effect of photosensitizer by light induced photosensitization. This cytotoxic effect of photosensitizer has been attributed to the production of singlet oxygen, superoxide ions, and hydroxyl radicals [22, 23]. These cytotoxic reactive oxygen species are primarily involved in cellular death of bacteria, which is either caused by the damage of their DNA or the lysis of their cell wall [20, 21]. Through the study of sterilization for* Staphylococcus aureus *using hematoporphyrin as photosensitizer, Bertoloni et al. [24] found that the cell membrane was the primary target. The electrophoresis of whole-cell protein analysis showed that cytoplasmic proteins had no change. But cell membrane proteins were changed, which would cause cell inactivation. The damage of DNA appeared as the break of single-stranded DNA and double-stranded DNA and the disappearance of plasmid superhelix fragment [25–27]. Kumar et al. [28] found that photosensitizer riboflavin could combine with nucleic acid of bacteria and further damage their DNA by the photochemical reactions. The hydroxyl generated by nucleic acid could result in the base damage and the increase of DNA degradation. Some studies also indicated that it was different to eliminate the Gram-positive bacteria and Gram-negative bacteria. This has been attributed to the complex cell-wall structure of Gram-negative species compared to Gram-positive ones with lesser photosensitizer and light penetrating the cell-wall structure [29].

In our study, it was found that the susceptibility of* Staphylococcus epidermidis* and* Staphylococcus aureus* for PDT was different. Although* Staphylococcus epidermidis* and* Staphylococcus aureus* belong to the* Staphylococcus* and are both Gram-positive bacteria, the survival rate of* Staphylococcus epidermis *was lower than that of* Staphylococcus aureus *after being irradiated by the same UV light parameters. The reason may be related to the formation of bacterial biofilm. Previous studies have shown that bacteria can form a multibacterial complex during growth, consisting of bacteria and their secreted extracellular polysaccharides, known as the bacterial biofilm (BBF) [30, 31]. BBF can protect the internal bacteria from bactericide and escape from the host immune killer, so BBF is more difficult to eradicate than isolated bacteria.* Staphylococcus epidermis *and* Staphylococcus aureus *are able to form BBF, but the ability of two bacteria to form bacterial biofilm is not the same. Compared to* Staphylococcus epidermis*,* Staphylococcus aureus* is more likely to form multibacterial complex, which resists penetration of photosensitizer or light into the inside. Hence, in order to achieve the same bactericidal efficacy, higher light energy or longer irradiation time could be required. Further research should be conducted to reveal their mechanism.

In conclusion, our study revealed that common pathogens of ocular surface could be effectively inactivated. However, because of the different susceptibility in PDT treatment, the irradiation parameters should be adjusted according to the different pathogenic bacteria. PDT treatment can prove to be effective for cornea infections. A further insight in PDT with* in vivo *experiment is required to ensure the effectiveness of the antimicrobial PDT.

## Figures and Tables

**Table 1:** Survival fraction of *Staphylococcus epidermis* and *Staphylococcus aureus* with different UV light energy in the UV-riboflavin group (irradiation time: 20 min).

Power density (mW/cm^2^)	Survival fraction of *S. epidermidis* (%)	Survival fraction of *S. aureus* (%)
0	100	100
1.065	55.1 ± 3.6	70.5 ± 2.7
2.474	43.5 ± 2.2	56 ± 2.3
5.273	38.1 ± 1.9	43.3 ± 1.7
7.299	28.3 ± 1.6	32.6 ± 1.5

**Table 2:** Survival fraction of *Staphylococcus epidermis* and *Staphylococcus aureus* under different UV irradiation time in the UV-riboflavin group (UV light energy: 5.273 mW/cm^2^).

Irradiation time (min)	Survival fraction of *S. epidermidis* (%)	Survival fraction of *S. aureus* (%)
0	100	100
5	47.5 ± 3.3	60.8 ± 4.3
10	43.5 ± 2.6	53.3 ± 3.0
20	38.1 ± 1.9	43.3 ± 1.7
30	6.2 ± 0.7	25.4 ± 1.4
